# Clinical Characteristics of Omicron SARS-CoV-2 Variant Infection After Non-mRNA-Based Vaccination in China

**DOI:** 10.3389/fmicb.2022.901826

**Published:** 2022-06-30

**Authors:** Qing-Lei Zeng, Yuan-Jun Lv, Xiao-Jing Liu, Zhi-Yong Jiang, Shuo Huang, Wei-Zhe Li, Zu-Jiang Yu

**Affiliations:** ^1^Department of Infectious Diseases, The First Affiliated Hospital of Zhengzhou University, Zhengzhou, China; ^2^Department of Respiratory Medicine, The First Affiliated Hospital of Zhengzhou University, Zhengzhou, China; ^3^Department of Neurology, The First Affiliated Hospital of Zhengzhou University, Zhengzhou, China; ^4^Department of Internal Medicine, The Fifth People’s Hospital of Anyang City, Anyang, China

**Keywords:** China, coronavirus disease 2019, omicron variant, severe acute respiratory syndrome coronavirus 2, vaccination, viral shedding

## Abstract

**Introduction:**

To date, little is known about the real-world protective role of Chinese inactivated and recombinant coronavirus disease 2019 (COVID-19) vaccines under the background of the long-term “Dynamic Zero COVID-19 Case” (i.e., no infection source) in China, especially when facing the widespread Omicron severe acute respiratory syndrome coronavirus 2 (SARS-CoV-2) variant infection.

**Methods:**

In this prospective, single-center cohort study, the clinical characteristics of post-vaccination Omicron SARS-CoV-2 variant infection were investigated in the initial largest outbreak of Omicron SARS-CoV-2 variant infection that occurred between the 8 January, 2022 and 29 January, 2022 in Anyang City, Henan Province, China. The primary endpoints were the rates of severe and critical diseases or death. The secondary endpoints were the SARS-CoV-2 shedding duration and length of hospitalization.

**Results:**

A total of 380 post-vaccination patients infected with the Omicron SARS-CoV-2 variant were enrolled. The median age was 18 (interquartile range [IQR] 17–35) years, 219 (57.6%) cases were female, and 247 (65.0%) cases were students. Before confirmation of Omicron SARS-CoV-2 variant infection, patients had 3 (IQR 2–4) days of dry cough (40.3%), nasal congestion (26.3%), and sore throat (26.3%). On admission, 294 (77.4%) cases had normal chest computerized tomography (CT) imaging. Additionally, only 5 (1.3%), 30 (7.9%), 4 (4/342, 1.2%), and 7 (7/379, 0.2%) patients had lymphocyte counts <800 per mm^3^, C-reactive protein levels >10 mg/L, lactate dehydrogenase levels ≥250 U/L, and D-dimer levels ≥0.5 mg/L on admission, respectively. During hospitalization, 308 (81.1%) and 72 (18.9%) were identified as mild and moderate cases, respectively, and no one progressed to severe and critical types, with a SARS-CoV-2 shedding period and length of hospital stay of 17 (IQR 12–22) and 19 (IQR 15–24) days, respectively.

**Conclusion:**

The current study found that approximately 80% of individuals infected with the Omicron SARS-CoV-2 variant were mild, approximately 20% of patients were moderate, and no severe, critical, or fatal cases were identified in a prospective cohort including 380 participants vaccinated with non-mRNA-based vaccines.

**Discussion:**

This study supports the consideration of policy adjustments and changes to prevent and control the Omicron-predominant COVID-19 in China and other regions with high SARS-CoV-2 vaccination rates.

## Introduction

From 8 to 29 January 2022, Anyang City of Henan Province, the hometown of Chinese oracle-bone inscriptions, initially experienced the largest outbreak of Omicron severe acute respiratory syndrome coronavirus 2 (SARS-CoV-2) variant infection in China, which was transmitted from the Tianjin Municipality (Xinhua News Agency (The Official State Press Agency of the People’s Republic of China), 2022). This outbreak took place in schools and mainly affected high school students and teachers as well as their families, with a total of 468 cases confirmed as Omicron coronavirus disease 2019 (COVID-19).

A small number (81 [17.3%]) of asymptomatic or relatively milder patients were hospitalized in several county-level hospitals, and a total of 387 (82.7%) “more severe” patients were transferred to The Fifth People’s Hospital of Anyang City, which was immediately and completely taken over by The First Affiliated Hospital of Zhengzhou University (one of largest tertiary and teaching hospital in China) after identification of several initial Omicron COVID-19 cases. Because the vast majority of patients were vaccinated with SARS-CoV-2 vaccines, the current study aimed to investigate the clinical characteristics of these patients with post-vaccination Omicron SARS-CoV-2 variant infection during the largest Omicron COVID-19 outbreak in China at the time.

## Materials and Methods

### Study Design

This study was a prospective, single-center cohort study. All patients were hospitalized in the designated COVID-19 hospital in Anyang City, i.e., The Fifth People’s Hospital of Anyang City in Henan Province, China. Notably, all the patients received health care service from the medical staff of the above-mentioned The First Affiliated Hospital of Zhengzhou University. We prospectively collected and analyzed the epidemiological, clinical, laboratory, virological, management, and outcome data of post-vaccination patients with submicron SARS-CoV-2 variant infection.

### Inclusion and Exclusion Criteria

This study was designed to include all the reachable patients hospitalized in the designated COVID-19 hospital in Anyang City, i.e., The Fifth People’s Hospital of Anyang City in Henan Province, China. Notably, because we intended to investigate the clinical characteristics of post-vaccination COVID-19 patients, we excluded only those patients who did not receive at least one dosage of a COVID-19 vaccine.

### SARS-CoV-2 Testing

The RT-PCR diagnostic reagents for SARS-CoV-2 infection were provided by Shanghai BioGerm Medical Biotechnology, China, and suspicious results were confirmed by using a different reagent, i.e., Guangzhou DAAN GENE Detection kit for 2019-nCoV, China. A positive result was defined as a cycle threshold of less than 40. The submicron SARS-CoV-2 variants were identified by both the Centers for Disease Control and Prevention of Anyang City and Henan Province using gene sequencing.

### Study Outcomes

The primary endpoints were the rates of severe disease, critical disease, and fatality. The secondary endpoints were the SARS-CoV-2 shedding duration and length of hospitalization. Disease severity classifications were based on the latest Chinese COVID-19 guidelines (National Health Commission and National Administration of Traditional Chinese Medicine, 2021). The SARS-CoV-2 shedding period indicates the duration from the first SARS-CoV-2 RNA-positive result to the first consecutive SARS-CoV-2 RNA-negative result. The criteria for hospital discharge (recovery) were based on the recovery of symptoms and signs, the negativity of SARS-CoV-2 RNA and the absorption of lung inflammation. SARS-CoV-2 RNA negativity was confirmed at least two times with an interval of more than 24 h between each test.

### Sample Size Estimation

We did not estimate the sample size before study initiation because we could not predict and control the total number of COVID-19 cases in the uncertain future at that time, and we intended to include all the reachable cases at study initiation. However, for the record and to be clear about the power of the sample size in the current study, we determined that a final sample size of 380 in this study could achieve 97.495% and 73.130% power to capture the outcomes that occurred in 1% and 0.1% of cases, respectively, with a significance level of 0.025 (one-tailed test).

### Statistical Analysis

Continuous variables were summarized as either the mean ± standard deviation or the median and range, as appropriate. The percentage of patients in each category was calculated for categorical variables. The percentages were compared between the two groups using the chi-square test. The Mann–Whitney U test was performed to compare continuous variables between the two groups. A binary logistic regression model was used to estimate the association between some potential factors and clinical features and classifications. The analyses were performed using SPSS software 25.0 for Windows (SPSS Inc., Chicago, IL). A two-sided *p* < 0.05 was considered significant. Sample size was estimated using inequality tests for one proportion by PASS 15.0 for Windows (NCSS statistical software, Kaysville, UT, United States).

## Results

### Vaccination and Demographic Characteristics

Among the 387 Omicron SARS-CoV-2 variant-infected patients hospitalized in The Fifth People’s Hospital of Anyang City, 380 (98.2% [380/387]) were vaccinated with at least one dosage of COVID-19 vaccine, and the remaining 7 (1.8% [7/387]) mild/moderate patients were unvaccinated, including five children less than 3 years old that were not approved by the authorities. Among the 380 vaccinated patients, 369 (97.1% [369/380]) were fully vaccinated and confirmed to have COVID-19 at 143.0 (interquartile range [IQR] 135.0–168.0) days after full vaccination (Table 1). The median age was 18 (IQR 17–35) years, 219 (57.6%) were female, and 247 (65.0%) were students (Table 2). A total of 45 (11.8%) patients had coexisting disorders (Table 2).

**Table 1:** Vaccination characteristics of patients upon admission.

COVID-19 vaccination	Patients (*n* = 380)
Full inactivated vaccination (two doses)	355 (93.4)
Sinovac Coronavac	153 (40.3)
Sinopharm BBIBP-CorV	50 (13.2)
Sinovac Coronavac + Sinopharm BBIBP-CorV	152 (40.0)
Additional one booster dose (three doses totally)	10 (2.6)
Full recombinant vaccination (CHO cell, ZF2001, three doses)	11 (2.9)
Full recombinant vaccination (Adenovirus Type 5 Vector, one dose)	3 (0.8)
Partial vaccination	11 (2.9)
Inactivated vaccines	8 (2.1)
Recombinant vaccine (CHO cell, ZF2001)	3 (0.8)
**Time from full vaccination to COVID-19 confirmation—days**	143.0 (135.0–168.0)
**Time from partial vaccination to COVID-19 confirmation—days**	145.0 (120.0–211.0)

Data are presented as the median (interquartile range) or *n* (%). COVID-19 denotes coronavirus disease 2019.

**Table 2:** Demographic characteristics of patients on admission.

Characteristics	Patients (*n* = 380)
**Age**	
Median—years	18.0 (17.0–35.0)
3–14	26 (6.8)
15–49	299 (78.7)
50–64	35 (9.2)
≥65	20 (5.3)
**Female sex**	219 (57.6)
**Occupation**	
Student	247 (65.0)
Agricultural worker	95 (25.0)
Teacher or employee	31 (8.2)
Child	7 (1.8)
**Smoking history**	10 (2.6)
**Alcohol drinking history**	5 (1.3)
**Coexisting disorder (condition)**	
Any	45 (11.8)
Hypertension	15 (3.9)
Diabetes	5 (1.3)
Hepatitis B infection^†^	5 (1.3)
Coronary heart disease	4 (1.1)
Thyroid disease	3 (0.8)
Cerebrovascular disease	3 (0.8)
Cancer (resected)	2 (0.5)
Hepatitis C infection^‡^	1 (0.3)
Chronic bronchitis	1 (0.3)
Bronchiectasis	1 (0.3)
Epilepsia	1 (0.3)
Anaphylactoid purpura	1 (0.3)
Pregnancy	1 (0.3)
Hearing loss	1 (0.3)
Arthritis	1 (0.3)
Mania	1 (0.3)
Down’s syndrome	1 (0.3)
Myasthenia gravis	1 (0.3)
Urticaria	1 (0.3)
Renal calculus	1 (0.3)

Data are presented as the median (interquartile range) or *n* (%).

† The presence of hepatitis B infection was defined as a positive result for hepatitis B surface antigen with or without elevated levels of alanine or aspartate aminotransferase.

‡ The presence of hepatitis C infection was defined as a positive result for anti-hepatitis C virus (HCV) antibody; this patient refused to test for the HCV RNA level.

### Clinical Characteristics on Admission

**Figure 1:**
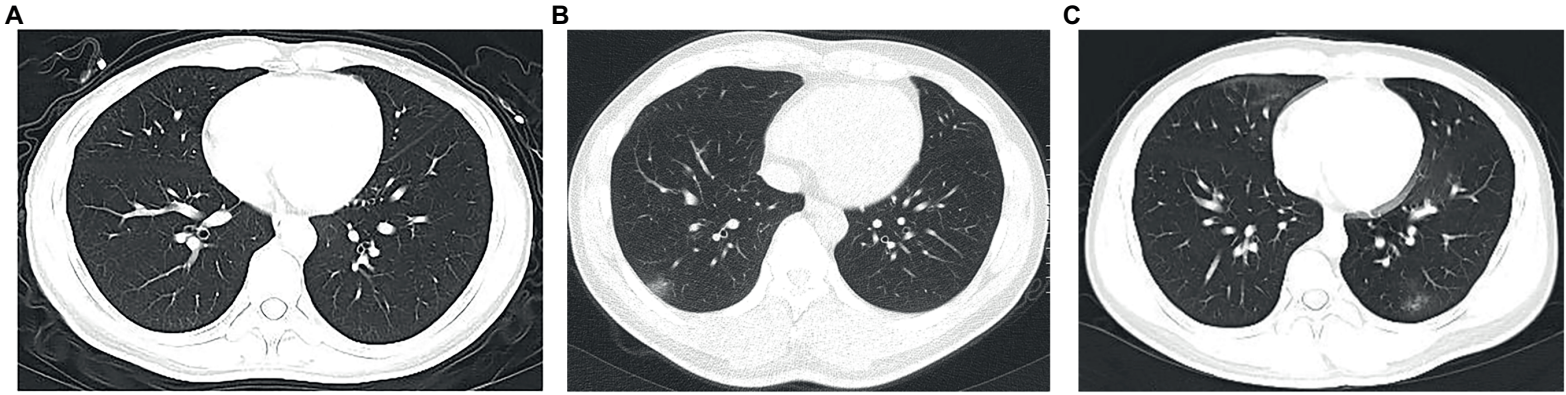
Chest computerized tomography images of patients with Omicron SARS-CoV-2 variant infection after inactivated and recombinant vaccinations in China. **(A)** Normal chest computerized tomography images. **(B)** Ground-glass opacity in left lung. **(C)** Mildly bilateral ground-glass opacity and patchy shadows.

Before COVID-19 confirmation, patients had a median of 3 (IQR 2–4) days of dry cough (153 [40.3%]), nasal congestion (100 [26.3%]), sore throat (100 [26.3%]), sputum production (88 [23.2%]), fever (86 [22.6%]), and runny nose (27 [7.1%]) (Table 3). On admission, 294 (77.4%) patients had normal findings on chest computerized tomography scan, and only 7 (1.8%) had multiple mottling and ground-glass opacities (Table 4; Figure 1). Notably, only 5 (1.3%), 30 (7.9%), 4 (4/342, 1.2%), and 7 (7/379, 0.2%) patients had lymphocyte counts <800 per mm^3^, C-reactive protein levels >10 mg/L, lactate dehydrogenase levels ≥250 U/L, and D-dimer levels ≥0.5 mg/L, respectively (Table 4).

**Table 3:** Clinical characteristics of patients on admission.

Symptoms and signs on admission	Patients (*n* = 380)
Any	344 (90.5)
Dry cough	153 (40.3)
Nasal congestion	100 (26.3)
Sore throat	100 (26.3)
Sputum production	88 (23.2)
Fever	86 (22.6)
38.0°C–38.9°C	13 (3.4)
≥39.0°C	2 (0.5)
Runny nose	27 (7.1)
Fatigue	13 (3.4)
Headache	9 (2.4)
Chest pain	5 (1.3)
Shortness of breath	4 (1.1)
Diarrhea	3 (0.8)
Nausea	2 (0.5)
Myalgia or arthralgia	1 (0.3)
Abdominal pain	1 (0.3)
Hypogeusia	1 (0.3)
**Time from symptom or sign onset to COVID-19 confirmation—days**	3.0 (2.0–4.0)

Data are presented as the median (interquartile range) or *n* (%). COVID-19 denotes coronavirus disease 2019.

**Table 4:** Imaging and laboratory findings of patients upon admission.

Characteristics	Patients (*n* = 380)
**Chest CT findings on admission**	
Normal	294 (77.4)
Local patchy shadowing	56 (14.7)
Bilateral patchy shadowing	23 (6.1)
Multiple mottling and ground-glass opacity	7 (1.8)
**Laboratory findings on admission**	
White-cell count	
Median—per mm^3^	4925 (4053–6245)
>10,000 per mm^3^	3 (7.9)
<4,000 per mm^3^	89 (23.4)
Lymphocyte count	
Median—per mm^3^	1895 (1490–2395)
<800 per mm^3^	5 (1.3)
C-reactive protein	
Median—mg/L	1.9 (0.7–4.7)
≥10 mg/L	30 (7.9)
Procalcitonin	
<0.05 ng/ml	367 (96.6)
≥0.5 ng/ml	0 (0)
Lactate dehydrogenase	
Median—U/L	157.0 (139.8–179.0)
≥250 U/L	4/342 (1.2)
D-dimer	
Median—mg/L	0.04 (0.02–0.09)
≥0.5 mg/L	7/379 (0.2)
Platelet count	
Median—per mm^3^	205000 (173250–241500)
<100,000 per mm^3^	0 (0)
Hemoglobin—g/dl	14.2 (13.1–15.9)
Alanine aminotransferase	
Median—U/L	16.5 (12.0–26.0)
>40 U/L	44 (11.6)
Total bilirubin	
Median—μmol/L	11.1 (8.4–12.8)
>17.1 μmol/L	55 (14.5)
Creatine kinase ≥ 200 U/L	
Median—U/L	86.0 (64.0–117.0)
≥200 U/L	21/342 (6.1)
Creatinine	
Median—μmol/L	69 (62.0–79.0)
≥133 μmol/L	0 (0)

Data are presented as the median (interquartile range), *n* (%), or *n*/*N* (%), where *N* is the total number of patients with available data. CT denotes computerized tomography.

### Clinical Characteristics During Hospitalization

**Figure 2:**
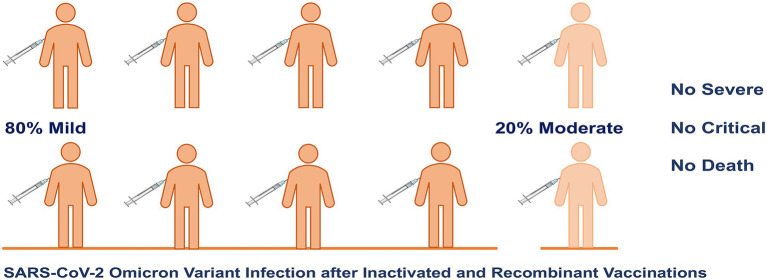
Clinical classification of Omicron SARS-CoV-2 variant infection after inactivated and recombinant vaccinations in China.

During hospitalization, 308 (81.1%) and 72 (18.9%) patients were identified as mild and moderate cases, respectively, and only 15 (3.9%) patients needed oxygen therapy (Table 5; Figure 2). Severe complications related to COVID-19 were not identified during hospitalization. However, 3 (0.8%) patients were admitted to the intensive care unit (ICU) due to coronary heart disease and acute pancreatitis; fortunately, no patients died due to these disorders (Table 5).

**Table 5:** Clinical characteristics of patients during hospitalization.

Characteristics	Patients (*n* = 380)
**Clinical classification during hospitalization**	
Mild cases	308 (81.1)
Moderate cases	72 (18.9)
Severe cases	0 (0)
Critical cases	0 (0)
**Complications during hospitalization**	
Coronary heart disease	2 (0.5)
Acute pancreatitis	1 (0.3)
Septic shock	0 (0)
Acute respiratory distress syndrome	0 (0)
**Treatments during hospitalization**	
Symptomatic therapy	160 (42.1)
Oxygen therapy	15 (3.9)
Transfer to intensive care unit^†^	3 (0.8)
Mechanical ventilation	0 (0)

Data are presented as *n* (%).

† COVID-19 was not the cause for transfer to the intensive care unit.

### Clinical Outcomes

As of 27 February 2022, no one progressed to severe and critical types, and all 380 (100%) patients were recovered and discharged, with a SARS-CoV-2 shedding period and length of hospital stay of 17 (IQR 12–22) and 19 (IQR 15–24) days, respectively (Table 6).

**Table 6:** Patient clinical outcomes.

Characteristics	Patients (*n* = 380)
**Clinical outcomes**	
Progressed to severe or critical type during hospitalization	0 (0)
Death	0 (0)
Discharge (recovery) from hospital	380 (100)
SARS-CoV-2 shedding period—days	17.0 (12.0–22.0)
Median length of hospital stay—days	19.0 (15.0–24.0)

Data are presented as the median (interquartile range) or *n* (%). SARS-CoV-2 denotes severe acute respiratory syndrome coronavirus 2.

### Clinical Outcomes in Patients With Various Vaccination Conditions

Given that no primary outcomes occurred in the current study, we only analyzed the potential differences in clinical classifications and secondary outcomes (i.e., the SARS-CoV-2 shedding period and length of hospital stay) among patients with various vaccination conditions. Notably, no significant differences for the clinical classifications were observed among patients with full inactivated vaccination, full recombinant vaccination, and partial vaccination (Table 7). Additionally, the ages were younger and the SARS-CoV-2 shedding periods were shorter in patients with full inactivated vaccination than in patients with full recombinant vaccination (Table 7).

**Table 7:** Clinical outcomes in patients with various vaccination conditions.

Parameters	Full inactivated vaccination (*n* = 355)	Full recombinant vaccination (*n* = 14)	Partial vaccination (*n* = 11)	*p*1 value	*p*2 value
Age—years	18.0 (17.0–31.0)	49.5 (39.5–63.8)	22.0 (18.0–38.0)	<0.001	0.316
Female sex	202 (56.9)	10 (71.4)	7 (63.6)	0.281	0.921
Mild cases	288 (81.1)	11 (78.6)	9 (81.8)	1.000	1.000
Moderate cases	67 (18.9)	3 (21.4)	2 (18.2)	1.000	1.000
SARS-CoV-2 shedding period—days	17.0 (12.0–22.0)	20.5 (17.8–26.3)	16.0 (9.0–25.0)	0.037	0.679
Median length of hospital stay—days	19.0 (15.0–24.0)	23.0 (19.0–28.3)	19.0 (15.0–26.0)	0.056	0.981

Data are presented as the median (interquartile range) or *n* (%). SARS-CoV-2 denotes severe acute respiratory syndrome coronavirus 2. *p*1: fully inactivated vaccination vs. fully recombinant vaccination; *p*2: fully inactivated vaccination + fully recombinant vaccination vs. partial vaccination.

## Discussion

More than 2 years have passed, and many situations have changed, including widespread SARS-CoV-2 vaccination and SARS-CoV-2 virulence. The clinical characteristics of Omicron SARS-CoV-2 infection are now different from those of the previous original SARS-CoV-2 infection (Chen et al., 2020; Guan et al., 2020; Zeng et al., 2020a,b). Generally, under the new circumstances, this Chinese, initial largest outbreak of Omicron SARS-CoV-2 infection tells us that patients with post-vaccination Omicron SARS-CoV-2 infection become sick faster, had milder symptoms and signs, and a much lower possibility of progressing to severe or critical diseases.

Notably, the current study showed that the clinical features of COVID-19 are evolving, not only including the shorter median incubation period and changed frequencies of clinical symptoms and signs but also including the rates of positive radiologic findings and the disease severity of COVID-19. For example, 88.7% of original SARS-CoV-2-infected patients had fever (Guan et al., 2020), and currently, only 22.6% (86/380) of patients with post-vaccination Omicron SARS-CoV-2 infection had fever. Additionally, before the large-scale SARS-CoV-2 vaccination, approximately 80% of COVID-19 patients were moderate cases (National Health Commission and National Administration of Traditional Chinese Medicine, 2021), and currently, 81.1% (308/380) of patients with post-vaccination Omicron SARS-CoV-2 infection are mild cases. Interestingly, a recent study reported that runny nose, headache, and fatigue are the most common symptoms of SARS-CoV-2 Omicron infection (Iacobucci, 2021); however, in the current study, dry cough (40.3%), nasal congestion (26.3%), and sore throat (26.3%) were the top three symptoms, which indicated that the clinical features of Omicron COVID-19 may be slightly different between nations with some different or unknown circumstances and backgrounds, such as vaccination rates and types. Notably, we intended to analyze the potential associations between the demographic characteristics and clinical features of the patients, however, no significant results were observed, and we suspect this is mainly because our cohort including overwhelmingly young populations, which may decrease the heterogeneity.

Recently, an increasing number of studies have indicated that the SARS-CoV-2 Omicron variant attenuates replication and pathogenicity and may weaken COVID-19 vaccine protection (Callaway, 2021; Cao et al., 2022; Shuai et al., 2022; Yu et al., 2022; Zhao et al., 2022), and the Omicron breakthrough infections in the current study are in accordance with those studies. However, the mild or moderate diseases of Omicron COVID-19 cases as well as the fact that no patients were admitted to the ICU or died due to COVID-19 may be attributed to the vaccinations to some extent. Notably, we analyzed the potential risk factor for patients with moderate disease, it is found that age is the only risk factor in the univariate regression model, and patient ≥50 years is associated with 5.4 (95% confidence interval: 2.9–10.1, *p* < 0.001) times of risk for progressing to moderate disease compared to patient <50 years. In addition, no coexisting disorder was identified as the risk factor for developing to moderate disease, even in the univariate regression model, and the prevalence of each coexisting disorder is low in young populations (Table 2, from 0.3% to 3.9%) may contribute to this result.

The current study has limitation. First, we did not include all 468 Omicron COVID-19 patients for analysis. This is because the other 81 asymptomatic or relatively milder patients were hospitalized in several county-level hospitals. However, more than 80% (380/468 [81.2%]) of the “more severe” patients were enrolled in the current study and may be even more representative, and it is not difficult to imagine that the clinical symptoms and disease severity of patients will be milder than the current report after the addition of these 81 asymptomatic or relatively milder cases. Second, we did not have follow-up data after these patients were discharged from hospital. This is because the overall number of COVID-19 patients in China is relatively small, and patients’ personal details and contact information are kept confidential to avoid discrimination after discharging or recovery, so follow-up is generally not possible, not only for our study, but also for many other real-world COVID-19 studies in China. Currently, all the 380 patients have been discharged for more than 2 months, and we are almost certain that none of these patients have been re-infected with SARS-CoV-2, as only a few imported cases have been reported from Anyang City over the past 2 months.

To date, little is known about the real-world protective role of Chinese inactivated and recombinant COVID-19 vaccines against the background of the long-term “Dynamic ZERO COVID-19 Case” (i.e., no infection source) in China. Conclusively, in combination with the nature of the SARS-CoV-2 Omicron variant, the widespread SARS-CoV-2 vaccinations, and our findings that all Omicron COVID-19 cases are mild and moderate with no severe, critical, or deceased cases, this study supports the consideration of policy adjustments and changes to prevent and control the Omicron-predominant COVID-19 in China and other regions with high SARS-CoV-2 vaccination rates.

## Data Availability Statement

The original contributions presented in the study are included in the article/supplementary material, and further inquiries can be directed to the corresponding authors.

## Ethics Statement

The study protocol was approved by the Institutional Review Commission of The First Affiliated Hospital of Zhengzhou University (no. 2020-KY-116). Written informed consent to participate in this study was provided by the participants’ legal guardian/next of kin.

## Author Contributions

Q-LZ, Y-JL, and X-JL contributed equally to this work. Q-LZ and Z-JY contributed to the study concept, design, drafting, and critical review of the manuscript. Q-LZ, Y-JL, X-JL, Z-YJ, SH, and W-ZL contributed to the data collection, interpretation, and analysis. All authors contributed to the article and approved the submitted version.

## Funding

This study was supported by the National Natural Science Foundation of China (81970517). The National Natural Science Foundation of China had no role in the design and conduct of the study; collection, management, analysis, and interpretation of the data; preparation, review, or approval of the manuscript; or decision to submit the manuscript for publication.

## Conflict of Interest

The authors declare that the research was conducted in the absence of any commercial or financial relationships that could be construed as a potential conflict of interest.

## Publisher’s Note

All claims expressed in this article are solely those of the authors and do not necessarily represent those of their affiliated organizations, or those of the publisher, the editors and the reviewers. Any product that may be evaluated in this article, or claim that may be made by its manufacturer, is not guaranteed or endorsed by the publisher.
